# Analyzing Gluten Content in Various Food Products Using Different Types of ELISA Test Kits

**DOI:** 10.3390/foods10010108

**Published:** 2021-01-06

**Authors:** Ja Myung Yu, Jae Hoon Lee, Jong-Dae Park, Yun-Sang Choi, Jung-Min Sung, Hae Won Jang

**Affiliations:** Korea Food Research Institute, 245 Nongsaengmyeong-ro, Iseo-myeon, Wanju-gun, Jeollabuk-do 55365, Korea; j.m.yu@kfri.re.kr (J.M.Y.); Leejaehoon@kfri.re.kr (J.H.L.); jdpark@kfri.re.kr (J.-D.P.); kcys0517@kfri.re.kr (Y.-S.C.); jmsung@kfri.re.kr (J.-M.S.)

**Keywords:** gluten-free, gluten analysis, ELISA, sandwich method, R5 antibody, G12 antibody, celiac disease

## Abstract

Gluten is an insoluble protein produced when glutelins and prolamins, which are found in grains such as wheat, barley, and oats, combine to form an elastic thin film. This dietary gluten can cause severe contraction of the intestinal mucous membrane in some people, preventing nutrient absorption. This condition, called celiac disease (CD), affects approximately 1% of the world’s population. The only current treatment for patients with CD and similar diseases is lifelong avoidance of gluten. To analyze the gluten content in food, various enzyme-linked immunosorbent assay (ELISA) tests are currently used. In this study, the gluten content in various food products was analyzed using different kinds of ELISA test kits. For gluten-free food, three different ELISA test kits mostly yielded values below the limit of detection. However, gluten was detected at 24.0–40.2 g/kg in bread, 6.5–72.6 g/kg in noodles, and 23.0–86.9 g/kg in different powder food samples. A significant difference (*p* < 0.05) in gluten content was observed for these gluten-containing food products. Reproducibility issues suggest that it is necessary to use several ELISA kits for the accurate detection and quantification of gluten in various food products rather than using one ELISA kit.

## 1. Introduction

Gluten is a storage protein found in barley, rye, wheat, and their hybrids [1]. The solubility of gluten differs depending on the degree of aggregation. The monomeric protein dissolves in alcohol, and the polymeric forms dissolve in alcoholic solutions under conditions of reduced aggregation [2]. According to its chemical composition or solubility, gluten can be divided into acid-/alkali-soluble glutelin and alcohol-soluble prolamin groups [3,4]. Glutenin in the glutelin group is found in wheat [5]. Gliadin, hordein, secalin, and avenin of the prolamin group are respectively found in wheat, barley, rye, and oats. The prolamin content in gluten is generally determined to be approximately 50% [6,7].

Celiac disease (CD) can be classified as a chronic immune-mediated inflammatory pathology of the small intestine caused by dietary gluten [8]. When CD patients consume gluten-containing food, an autoimmune reaction occurs. The disease causes terrible contraction of the intestinal mucosa, preventing nutrient absorption, and its most common symptoms are diarrhea, anemia, fatigue, and growth retardation [9,10]. Thus, it is essential to maintain a gluten-free diet among these patients, as even trace levels of gluten can damage the mucosal membrane of the small intestine [11]. CD affects approximately 1% of the world’s population. The extensive use of yeast and refined grains is one of the most recent causes of such trends [8]. Currently, the only treatment for CD and most other related disorders is lifelong avoidance of gluten in the diet [12,13]. With the increase in the prevalence and the awareness of CD, the gluten-free food industry recorded a 136% growth rate from 2013 to 2015 [14,15].

To ensure the safety of gluten-free food for CD patients, they must adhere to a gluten content threshold [16]. According to the Codex Alimentarius Commission (CODEX STAN 118-1979), the Commission Implementing Regulation (EU 828/2014), and the U.S. Food and Drug Administration (FR Doc. 2013-18813), products labeled “gluten-free” must comply with gluten levels of less than 20 mg/kg [17,18,19]. Products considered “food specifically processed to reduce gluten content” and “low gluten-level” must comply with gluten levels between 20 and 100 mg/kg [20]. Because these values are defined, it is important to provide analytical tools that can accurately and precisely quantify gluten content in various food products [21].

Currently, the Codex Alimentarius Commission proposes enzyme-linked immunosorbent assay (ELISA), as an analytical method, to achieve gluten-free product labeling [22]. This technique includes sandwich methods and competitive polyclonal methods. Most commercial ELISA test kits are based on monoclonal antibodies (R5, Skerritt, G12, and α20) [23,24,25,26]. However, ELISA is very expensive, and the reproducibility of the results varies depending on the ELISA test kit type. Differences have been noted in the affinity for gliadin and glutenin of the R5 and the 401/21 antibodies across test kits [27]. Differences have also been observed between values obtained using R5 sandwich and competitive methods for gluten-containing cereals [22]. The effect of food matrices on the detection of gluten is reflected in the differences in recovery [28]. Therefore, whether ELISA is a precise and accurate method to measure gluten content in food remains unclear [29,30]. Furthermore, although many studies have been conducted to confirm the reproducibility of ELISA kits, most of these were performed using wheat flour, and confirmatory studies using a variety of food products on the market are insufficient.

In this study, ELISA methods using R5 and G12 antibodies—against secalin from rye and gliadin from wheat, respectively—[25,31] proposed by the Codex Alimentarius Commission as temporary authorized analytical methods were compared, and calibration was performed for the quantitative analysis of gluten in various food products on the market. The results of the qualitative analysis were compared with those of the quantitative analysis.

## 2. Materials and Methods

### 2.1. Materials

Alcoholic beverages, bread, noodles, powdered food items, and snacks, listed in Table 1, were purchased from a local market (Wanju, Korea). Each sample was homogenized in a blender. Samples were stored at −20 °C until analysis.

Gluten in various food products was analyzed using three different sandwich ELISA test kits, as shown in Table 2. The RIDASCREEN Gliadin test kit (R-Biopharm AG, Darmstadt, Germany), the Veratox for Gliadin R5 test kit (Neogen, Lansing, MI, USA.), and the AgraQuant Gluten G12 test kit (Romer Labs, Runcorn, U.K.) were used for quantitative analysis. Using the qualitative test kit AgraStrip Gluten G12 test kit (Romer Labs, Runcorn, UK), the gluten content was analyzed and compared with the results of quantitative analysis. The analytical method for gluten detection and analysis followed protocols provided by each test kit manufacturer. All food samples were analyzed in triplicate.

### 2.2. Quantitative Analysis of Gluten Using RIDASCREEN Gliadin Test

The analytical protocol followed the ELISA test kit manufacturer’s instructions precisely. The sample (0.5 g) was placed into a 50 mL centrifuge tube and incubated for 40 min at 50 °C by adding 2.5 mL of the cocktail solution. Subsequently, 80% ethanol (7.5 mL) was added and mixed for 60 min to extract gluten. Samples were centrifuged for 10 min at 2500× *g* at room temperature. Three independent extraction procedures for each food sample were performed with triplicate measurements.

Next, 100 μL of each blank, standard, and sample solution was added into the wells and incubated for 30 min at room temperature, after which the standard and the sample solutions were removed from the wells. All wells were washed with a wash buffer three times. Thereafter, 100 μL of the conjugate was added to the wells and incubated for 30 min. Next, the conjugate was removed, and the wells were washed three times. Thereafter, 50 μL of the substrate and 50 μL of chromogen were added to each well and incubated for 30 min in the dark. Finally, 100 μL of the stop solution was added to measure the absorbance at 450 nm.

### 2.3. Quantitative Analysis of Gluten Using Veratox for Gliadin R5 kit

For samples that were not subjected to the heat treatment process, 1.0 g of the sample and the extraction additive were placed in a centrifuge tube (Fisher Scientific, Pittsburgh, PA, USA). Subsequently, 10 mL of 60% ethanol was added and mixed for 10 min. After centrifugation for 10 min at 2500× *g*, 100 μL of the upper layer of the extract was put into the tube, and 4.9 mL of the sample extract dilution solution (phosphate buffered saline, PBS, Sigma, MO, USA) was added to dilute each sample at a 1:50 ratio. The diluted samples were analyzed within 2–3 h.

Samples (0.25 g) for the heat treatment process were put into a tube, and 2.5 mL of the cocktail solution was added (if the samples contained buckwheat, chestnut, and tannin, an extraction additive was added to prevent disruption of analysis due to polyphenols) [22]. The mixture was homogenized for 30 s and incubated at 50 °C for 10 min. Three minutes later, 7.5 mL of 80% ethanol was added, and the mixture was shaken for 1 h. After extraction, the sample was centrifuged for 10 min at 2500× *g*. PBS (2.3 mL) was added to 200 μL of the upper layer to dilute the sample at a 1:12.5 ratio.

Approximately 150 μL of a blank, standard, and samples was injected into the red-marked mixing well, and 100 μL of each aliquot was moved into the antibody-coated well, and then incubation was performed for 10 min, after which the red-marked mixing well was removed from the plate. The standard and the sample solution in the antibody-coated well were removed, and the wells were washed five times with a wash buffer. After adding 100 μL of the conjugate to the well, the plate was incubated for 10 min, and then the conjugate was removed and the well washed five times. Next, 100 μL of the substrate was added to the well and incubated for 10 min, and then 100 μL of the stop solution was added to measure the absorbance at 650 nm (VersaMax™ microplate ELISA reader, Molecular Device, CA, USA).

### 2.4. Quantitative Analysis of Gluten Using AgraQuant Gluten G12 kit

A 0.25 g specimen was placed in a tube, and 2.5 mL of an extension solution was added. The mixture was incubated at 50 °C for 40 min, and 80% ethanol (7.5 mL, Merch, Darmstadt, Germany) was added with a rotator for 60 min for extraction. The extracts were centrifuged for 10 min at 2000× *g*.

The wells were then washed with the wash buffer five times. Thereafter, 100 μL of the conjugate was added to each well and incubated for 20 min and then removed. The washing step was repeated five times. Next, 100 μL of the substrate was added to the well and incubated for 20 min in the dark. Finally, 100 μL of the stop solution was added to each well to measure the absorbance at 450 nm.

### 2.5. Qualitative Analysis of Gluten Using AgraStrip Gluten G12 kit

A 0.2 g sample was put into the extraction tube, and 2.5 mL of the extension buffer was added. About 100 μL of the extract was put into the dilution tube, and the dilution buffer was added up to the mark of 5 mg/kg. After dipping the test strip vertically, we waited for 45 s for the solution to rise to the flow level line. The test strip was removed from the tube, and the result was checked 10 min later. If a single blue line appeared in the result zone, it was considered a negative result, but if blue and red lines appeared, it was considered a positive result. If a positive reaction occurred at 5 mg/kg, the dilution buffer was added up to the 10 mg/kg and 20 mg/kg marks, and the same process was repeated.

### 2.6. Statistical Analysis

Statistical analysis was performed using IBM SPSS Statistics 23 (IBM, Armonk, NY, USA). Data were analyzed by one-way ANOVA and Duncan’s multiple range test for investigating significant differences (*p* < 0.05).

## 3. Results

### 3.1. Calibration of ELISA Test Kits

Gluten usually includes gliadin and glutenin at a ratio of 1:1 [32,33]. In the case of the RIDASCREEN gliadin kit and the Veratox for gliadin R5 kit, twice the quantitation value of gliadin was calculated as the approximate content of gluten. The AgraQuant Gluten G12 kit indicates the detected gluten content. For quantitative analysis, standard calibration curves of five points were obtained using each ELISA kit. The limit of detection (LOD) and the limit of quantitation (LOQ) were validation data specified by the manufacturers. The results and data are shown in Table 3.

Calibration of RIDASCREEN gliadin kit based on the R5 antibody was performed as follows. Linearity was confirmed in the concentration range of 5‒40 µg/mL. The correlation coefficient for the linearity of the four points was R^2^ > 0.91. For the concentration range of 5‒80 µg/mL, the correlation coefficient for the quadratic of five points was R^2^ > 0.99. More than 1.0 mg/kg of gluten could be detected, and the LOQ was 5.0 mg/kg.

The linearity of Veratox for gliadin R5 kit based on the R5 antibody was found in the 5–40 µg/mL concentration range. The correlation coefficient of the four points for linearity was R^2^ > 0.99. In the case of the concentration range of 5‒80 µg/mL, the correlation coefficient for the quadratic of five points was R^2^ > 0.99. The LOD and the LOQ were estimated at 5.0 mg/kg, respectively.

The linearity of AgraQuant gluten G12 kit based on the G12 antibody was found in the 5–100 µg/mL concentration range. The correlation coefficient for the four points was *R*^2^ > 0.98. For the concentration range of 5‒200 µg/mL, the correlation coefficient for the quadratic of five points was *R*^2^ > 0.99. The estimated LOD and LOQ were 2.0 mg/kg and 4.0 mg/kg, respectively.

From the linearity results of the absorbance readings, a quadratic regression was used in all samples in this study.

### 3.2. Results of Qualitative Analysis of Gluten in Products

The qualitative analysis results using the AgraStrip Gluten G12 Kit are shown in Table 4. Gluten detection was performed for 21 types of food products. Among breads, only plain bread made of wheat flour gave a positive result. Among noodles, buckwheat soba, plain noodles, instant noodles, spaghetti noodles, and udon noodles tested positive. Among the powders, strong and soft wheat flour tested positive. All snacks tested negative. In total, only eight samples were found to contain gluten.

### 3.3. Results of Quantitative Analysis of Gluten in Gluten-Containing Products

**Figure 1:**
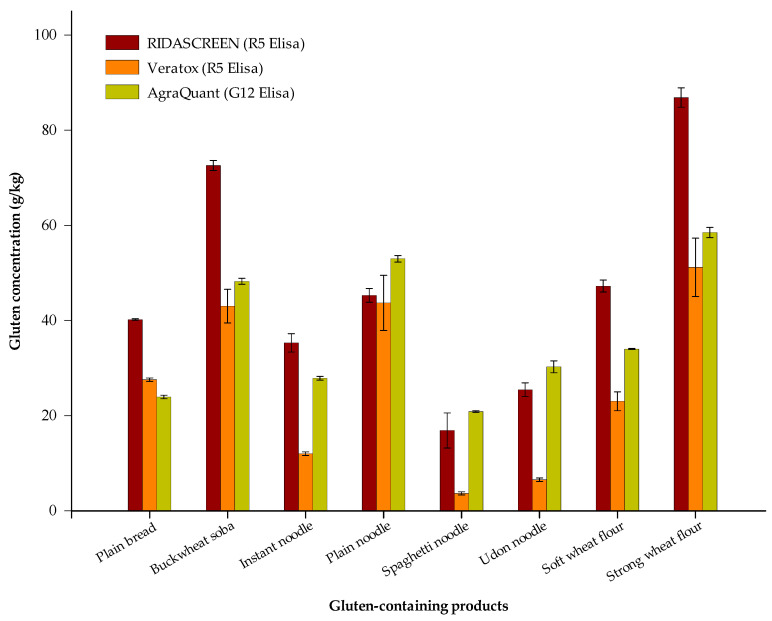
Gluten concentration in gluten-containing products measured using three types of sandwich ELISA.

The results of gluten content in eight samples containing gluten obtained using three sandwich ELISAs are shown in Figure 1. Gluten was detected in all the samples. Supplementary Table S1 shows the average and the relative standard deviations. In the case of powders, the highest content was 51.2–86.9 g/kg for strong wheat flour. Soft wheat flour was found to contain 23.0–47.3 g/kg of gluten. Flour is classified according to its gluten content. If the gluten content is high, it is classified as a strong flour; otherwise, it is classified as soft. Of the noodles, buckwheat soba showed the highest gluten content at 43.2–72.6 g/kg, followed by plain noodles (43.7–53.0 g/kg), instant noodles (12.0–35.3 g/kg), udon noodles (6.5–30.3 g/kg), and spaghetti noodles (3.7–20.9 g/kg). Plain bread gave a value of 24.0–40.2 g/kg. A significant difference (*p* < 0.05) was noted when quantification was undertaken using three different ELISA test kits for gluten-rich food.

### 3.4. Results of Gluten Content in Gluten-Free Products

The results of gluten content in 13 types of gluten-free samples are shown in Table 5. Bread, noodles, and snacks had values below the LOD or the LOQ. Among powder products, when analyzed using AgraQuant kit, a small amount (5.6 mg/kg) of gluten was detected only in green bean powder.

## 4. Discussion

In this study, the reproducibility of several commercial ELISA test kits for the quantification of gluten content was assessed. The RIDASCREEN and the Veratox test kits employ the affinity of the R5 antibody for gliadin, whereas AgraQuant employs that of the G12 antibody for gliadin. All three types of test kits were used in the sandwich method. For the RIDASCREEN gliadin test kit and the Veratox for Gliadin R5 test kit, the correlation coefficients for quadratic regression in the concentration range of 5‒80 ng/mL were *R*^2^ > 0.99 and *R*^2^ > 0.99, respectively. The LODs were 1.0 mg/kg and 5.0 mg/kg, respectively, and the LOQs were 5.0 mg/kg for both. For the AgraQuant Gluten G12 test kit, the correlation coefficient for quadratic in the concentration range of 5 to 200 ng/mL was *R*^2^ > 0.99. The LODs and the LOQs were 2.0 and 4.0 mg/kg, respectively. For the qualitative analysis of gluten using the AgraStrip Gluten G12 kit, the gluten-free food tested negative for gluten. Using three different ELISA test kits for quantitative analysis, the obtained results were mostly below the LOD. Some gluten-free powders, such as green bean powder, had gluten contents of 5.6 mg/kg. This may due to the contamination with flour during production. The plant where the powder production process is carried out also produces wheat flour, corn starch, green bean powder, and sugarcane powder, leading to a potential cross-contamination of the products. However, according to CODEX, European Commission Regulation, and the U.S. Food and Drug Administration, products with a gluten content of less than 20 mg/kg can be labeled “gluten-free” [18,19].

In contrast, the gluten contents of gluten-rich products were 23.0–86.9 g/kg for flour, 3.7–72.6 g/kg for noodles, and 24.0–40.2 g/kg for bread. For these gluten-rich products, when quantification was carried out using the three different ELISA test kits, a significant difference (*p* < 0.05) in gluten content was observed. This is due to differences in the antibody characteristics and the extract solutions of the ELISA test kits. Various food matrices remain difficult to analyze owing to either interference of antibody binding by the food matrix or cross-reactivity [22]. The results depend on the extraction method when using a cocktail solution containing 2-mercaptoethanol [34]. Several previous studies have reported similar results to ours. Scherf [35] reported that seven commercial ELISA test kits showed different gluten assay results in wheat products, especially gluten-free wheat. In addition, other studies using ELISA kits reported gluten contents higher than the stipulated threshold for gluten-free products. Likewise, Bruins Slot et al. [22] also reported similar results revealing that oat flour (a gluten-free labeled product) had a gluten content of more than 20 mg/kg and that the measurement error is large between the different commercial gluten kits used to measure the gluten content in foods. They concluded that it was due to the difference in the ability to extract gluten from the food matrix, the difference in the sensitivity of the used antibody, and in the reference material of the kits. As a result, the analysis of gluten content using such kits has some drawbacks, and thus an accurate analysis through a new analysis technique is required.

## 5. Conclusions

In summary, using three commercial ELISA test kits for measuring gluten content in various food products, calibration and quantitative analyses were conducted. The results showed that the reproducibility of the three kits was low. These kits had to accurately detect gluten in mg/kg units because the standard level for gluten-free products is 20 mg/kg. However, the experimental results confirmed an error in the g/kg units. Therefore, methods of extraction or device analysis that utilize precision analysis devices should be studied to ensure safe products for CD patients.

## Figures and Tables

**Table 1:** Food products used in analysis.

Type of Food	Product	Label
Bread	Black rice bread	Gluten-free
	Plain bread	Contains gluten
	White rice bread	Gluten-free
Noodles	Buckwheat soba	Contains gluten
	Cellophane noodle	Gluten-free
	Instant noodle	Contains gluten
	Plain noodle	Contains gluten
	Rice noodle	Gluten-free
	Spaghetti noodle	Contains gluten
	Udon noodle	Contains gluten
Powdered food items	Corn starch	Gluten-free
	Green bean powder	Gluten-free
	Potato starch	Gluten-free
	Rice flour	Gluten-free
	Soft wheat flour	Contains gluten
	Strong wheat flour	Contains gluten
	Sugar cane powder	Gluten-free
Snacks	Brown rice cereal	Gluten-free
	Brown rice snack	Gluten-free
	Corn cereal	Gluten-free
	Rice snack	Gluten-free

**Table 2:** Characteristics of commercial ELISA test kits.

Test Kit	Manufacturer	Format	Antibody	Target Protein
RIDASCREEN Gliadin	R-Biopharm	Sandwich	R5	Gliadin
Veratox for Gliadin R5	Neogen	Sandwich	R5	Gliadin
AgraQuant Gluten G12	Romer Labs	Sandwich	G12	Gluten
AgraStrip Gluten G12	Romer Labs	Lateral flow device (LFD)	G12	Gluten

**Table 3:** Linearity and sensitivity of sandwich ELISA test kits.

Test Kit	Linearity			Sensitivity	
	Linear Range (ng/mL)	Calibration Curve	R^2^	LOD (mg/kg)	LOQ (mg/kg)
RIDASCREEN gliadin kit	5–40	Linear	0.9175	1.0	5.0
	5–80	Quadratic	0.9953		
Veratox for gliadin R5	5–40	Linear	0.9966	5.0	5.0
	5–80	Quadratic	0.9988		
AgraQuant Gluten G12	5–100	Linear	0.9874	2.0	4.0
	5–200	Quadratic	0.9958		

LOD and LOQ mean limit of detection and limit of quantification, respectively.

**Table 4:** Results of qualitative analysis of gluten using AgraStrip Gluten G12 test kit.

Type of Food	Product	Test Result	
		5 mg/kg	10 mg/kg
Bread	Black rice bread	-	-
	Plain bread	+	+
	White rice bread	-	-
Noodles	Buckwheat soba	+	+
	Cellophane noodle	-	-
	Instant noodle	+	+
	Plain noodle	+	+
	Rice noodle	-	-
	Spaghetti noodle	+	+
	Udon noodle	+	+
Powder	Corn starch	-	-
	Green bean powder	-	-
	Potato starch	-	-
	Rice flour	-	-
	Soft wheat flour	+	+
	Strong wheat flour	+	+
	Sugar cane powder	-	-
Snacks	Brown rice cereal	-	-
	Brown rice snack	-	-
	Corn cereal	-	-
	Rice snack	-	-

If the result is positive, it is expressed as (+); otherwise, it is denoted as (-).

**Table 5:** Results for gluten-free products using three types of sandwich ELISA test kits.

Type of food	Product	Gluten Concentration (mg/kg)		
		RIDASCREEN (R5 ELISA)	Veratox (G5 ELISA)	AgraQuant (G12 ELISA)
Bread	Black rice bread	Below LOD	Below LOD	Below LOD
	White rice bread	Below LOD	Below LOD	Below LOD
Noodles	Cellophane noodle	Below LOD	Below LOD	Below LOD
	Rice noodle	Below LOD	Below LOD	Below LOD
Powder	Corn starch	Below LOD	Below LOD	Below LOD
	Green bean powder	Below LOQ	Below LOD	5.6 ± 0.4
	Potato starch	Below LOD	Below LOD	Below LOD
	Rice flour	Below LOD	Below LOD	Below LOD
	Sugar cane powder	Below LOD	Below LOD	Below LOD
Snacks	Brown rice cereal	Below LOD	Below LOD	Below LOD
	Brown rice snack	Below LOD	Below LOD	Below LOD
	Corn cereal	Below LOD	Below LOD	Below LOD
	Rice snack	Below LOQ	Below LOD	Below LOD

All values are denoted as mean ± standard deviation (*n* = 3).

## Data Availability

The data presented in this study are available in the article and the supplementary material.

## Supplementary Materials

The following are available online at https://www.mdpi.com/2304-8158/10/1/108/s1. Table S1, Concentration of gluten in gluten-containing products using three types of sandwich ELISA test kits.

## References

[1] Biesiekierski J.R. (2017). What is gluten?. J. Gastroenterol. Hepatol..

[2] Mena M.C., Lombardía M., Hernando A., Méndez E., Albar J.P. (2012). Comprehensive analysis of gluten in processed foods using a new extraction method and a competitive ELISA based on the R5 antibody. Talanta.

[3] Diaz-Amigo C., Popping B. (2013). Accuracy of ELISA detection methods for gluten and reference materials: A realistic assessment. J. Agric. Food Chem..

[4] Sharma G.M., Khuda S.E., Pereira M., Slate A., Jackson L.S., Pardo C., Williams K.M., Whitaker T.B. (2013). Development of an incurred cornbread model for gluten detection by immunoassays. J. Agric. Food Chem..

[5] Wieser H. (2007). Chemistry of gluten proteins. Food Microbiol..

[6] Koehler P., Schwalb T., Immer U., Lacorn M., Wehling P., Don C. (2013). AACCI approved methods technical committee report: Collaborative study on the immunochemical determination of intact gluten using an R5 sandwich ELISA. Cereal Food World.

[7] Scherf K.A., Poms R.E. (2016). Recent developments in analytical methods for tracing gluten. J. Cereal Sci..

[8] Gobbetti M., Pontonio E., Filannino P., Rizzello C.G., De Angelis M., Di Cagno R. (2018). How to improve the gluten-free diet: The state of the art from a food science perspective. Food Res. Int..

[9] Ribeiro M., Nunes F.M., Rodriguez-Quijano M., Carrillo J.M., Branlard G., Igrejas G. (2018). Next-generation therapies for celiac disease: The gluten-targeted approaches. Trends Food Sci. Technol..

[10] Goel G., Tye-Din J.A., Qiao S.-W., Russell A.K., Mayassi T., Ciszewski C., Sarna V.K., Wang S., Goldstein K.E., Dzuris J.L. (2019). Cytokine release and gastrointestinal symptoms after gluten challenge in celiac disease. Sci. Adv..

[11] Sapone A., Bai J.C., Ciacci C., Dolinsek J., Green P.H., Hadjivassiliou M., Kaukinen K., Rostami K., Sanders D.S., Schumann M. (2012). Spectrum of gluten-related disorders: Consensus on new nomenclature and classification. BMC Med..

[12] Lynch K.M., Coffey A., Arendt E.K. (2018). Exopolysaccharide producing lactic acid bacteria: Their techno-functional role and potential application in gluten-free bread products. Food Res. Int..

[13] Bascunan K.A., Vespa M.C., Araya M. (2017). Celiac disease: Understanding the gluten-free diet. Eur. J. Nutr..

[14] Reilly N.R. (2016). The gluten-free diet: Recognizing fact, fiction, and fad. J. Pediatr..

[15] Morreale F., Angelino D., Pellegrini N. (2018). Designing a score-based method for the evaluation of the nutritional quality of the gluten-free bakery products and their gluten-containing counterparts. Plant Foods Hum. Nutr..

[16] Lacorn M., Scherf K., Uhlig S., Weiss T. (2016). Determination of gluten in processed and nonprocessed corn products by qualitative R5 immunochromatographic dipstick: Collaborative study, first action 2015.16. J. AOAC Int..

[17] Hochegger R., Mayer W., Prochaska M. (2015). Comparison of R5 and G12 antibody-based ELISA used for the determination of the gluten content in official food samples. Foods.

[18] CODEX S. (2008). STAN 118-1979. Standard for Foods for Special Dietary Use for Persons Intolerant to Gluten.

[19] Commission E. (2014). Commission implementing regulation (EU) No 828/2014, requirements for the provision of information to consumers on the absence or reduced presence of gluten in food. Off. J. Eur. Union L..

[20] Verma A.K., Gatti S., Galeazzi T., Monachesi C., Padella L., Baldo G.D., Annibali R., Lionetti E., Catassi C. (2017). Gluten contamination in naturally or labeled gluten-free products marketed in Italy. Nutrients.

[21] Bugyi Z., Török K., Hajas L., Adonyi Z., Popping B., Tömösközi S. (2013). Comparative study of commercially available gluten ELISA kits using an incurred reference material. Qual. Assur. Saf. Crop.

[22] Bruins Slot I.D., Bremer M.G., van der Fels-Klerx I., Hamer R.J. (2015). Evaluating the performance of gluten ELISA test kits: The numbers do not tell the tale. Cereal Chem..

[23] Sharma G.M. (2012). Immunoreactivity and detection of wheat proteins by commercial ELISA kits. J. AOAC Int..

[24] Skerritt J.H., Hill A.S. (1990). Monoclonal antibody sandwich enzyme immunoassays for determination of gluten in foods. J. Agric. Food Chem..

[25] Moron B., Bethune M.T., Comino I., Manyani H., Ferragud M., Lopez M.C., Cebolla Á., Khosla C., Sousa C. (2008). Toward the assessment of food toxicity for celiac patients: Characterization of monoclonal antibodies to a main immunogenic gluten peptide. PLoS ONE.

[26] Spaenij-Dekking E., Kooy-Winkelaar E., Nieuwenhuizen W., Drijfhout J., Koning F. (2004). A novel and sensitive method for the detection of T cell stimulatory epitopes of α/β-and γ-gliadin. Gut.

[27] Allred L.K., Ritter B.W. (2010). Recognition of gliadin and glutenin fractions in four commercial gluten assays. J. AOAC Int..

[28] Scharf A., Kasel U., Wichmann G., Besler M. (2013). Performance of ELISA and PCR methods for the determination of allergens in food: An evaluation of six years of proficiency testing for soy (Glycine max L.) and wheat gluten (Triticum aestivum L.). J. Agric. Food Chem..

[29] Lupo A., Roebuck C., Walsh A., Mozola M., Abouzied M. (2013). Validation study of the Veratox R5 Rapid ELISA for detection of gliadin. J. AOAC Int..

[30] Koerner T.B., Abbott M., Godefroy S.B., Popping B., Yeung J.M., Diaz-Amigo C., Roberts J., Taylor S.L., Baumert J.L., Ulberth F. (2013). Validation procedures for quantitative gluten ELISA methods: AOAC allergen community guidance and best practices. J. AOAC Int..

[31] Kanerva P.M., Sontag-Strohm T.S., Ryöppy P.H., Alho-Lehto P., Salovaara H.O. (2006). Analysis of barley contamination in oats using R5 and ω-gliadin antibodies. J. Cereal Sci..

[32] Barak S., Mudgil D., Khatkar B. (2013). Relationship of gliadin and glutenin proteins with dough rheology, flour pasting and bread making performance of wheat varieties. LWT Food Sci. Technol..

[33] Don C., Halbmayr-Jech E., Rogers A., Koehler P. (2014). AACCI Approved Methods Technical Committee report: Collaborative study on the immunochemical quantitation of intact gluten in rice flour and rice-based products using G12 sandwich ELISA. Cereal Food World.

[34] Geng T., Westphal C.D., Yeung J.M. (2008). Detection of gluten by commercial test kits: Effects of food matrices and extraction procedures. ACS Symp. Ser..

[35] Scherf K.A. (2017). Gluten analysis of wheat starches with seven commercial ELISA test kits—Up to six different values. Food Anal. Meth..

