# Supplementary material for: Analyzing Gluten Content in Various Food Products Using Different Types of ELISA Test Kits
Source: Foods. 2021 Jan 6;10(1):108. doi: 10.3390/foods10010108 (PMC7825509; doi:10.3390/foods10010108)
Supplement: Supplementary file 1 [file foods-10-00108-s001.pdf]

Supplementary File

Table S1. Concentration of gluten in gluten-containing products using three types of sandwich ELISA test kits

Type of Food	Product	Gluten Concentration of Samples (g/kg)		
		**RIDASCREEN (R5 ELISA)**	**Veratox (G5 ELISA)**	**AgraQuant (G12 ELISA)**
Bread	Plain bread	40.2 ± 0.1 [a]	27.6 ± 0.4 [b]	24.0 ± 0.4 [c]
Noodles	Buckwheat soba	72.6 ± 1.0 [a]	43.2 ± 3.5 [c]	48.3 ± 0.6 [b]
	Instant noodle	35.3 ± 1.9 [a]	12.0 ± 0.4 [c]	27.9 ± 0.4 [b]
	Plain noodle	45.3 ± 1.5 [b]	43.7 ± 5.8 [b]	53.0 ± 0.7 [a]
	Spaghetti noodle	16.9 ± 3.7 [a]	3.7 ± 0.3 [b]	20.9 ± 0.2 [a]
	Udon noodle	25.5 ± 1.4 [b]	6.5 ± 0.4 [c]	30.3 ± 1.2 [a]
Powder	Soft wheat flour	47.3 ± 1.3 [a]	23.0 ± 2.0 [c]	34.0 ± 0.1 [b]
	Strong wheat flour	86.9 ± 2.0 [a]	51.2 ± 6.1 [b]	58.5 ± 1.1 [b]

All values are denoted as mean ± standard deviation (n = 3). [a–c] within a row, different letters represent significantly different values ($p < 0.05$).
